# Even chained acylcarnitines predict long-term cardiovascular prognosis in patients with chest pain and non-obstructive coronary artery disease

**DOI:** 10.1016/j.ijcrp.2022.200134

**Published:** 2022-05-17

**Authors:** Silje Kjellevold Storesund, Iman Karaji, Elin Strand, Asbjørn Svardal, Mai Tone Lønnebakken, Rolf Kristian Berge, Gard Frodahl Tveitevåg Svingen, Ottar Kjell Nygård, Eva Ringdal Pedersen

**Affiliations:** a Department of Heart Disease, Haukeland University Hospital, Bergen, Norway; b Department of Clinical Science, University of Bergen, Bergen, Norway; c Department of Immunology and Transfusion Medicine, Haukeland University Hospital, Bergen, Norway

**Keywords:** Acylcarnitines, Non-obstructive coronary artery disease, Outcomes, Metabolism

## Abstract

**Background:**

Acylcarnitines are essential for mitochondrial fatty acid oxidation. Earlier studies suggest that impaired energy metabolism may be implicated in the pathogenesis of microvascular angina. We explored metabolites from the carnitine pathway as predictors of cardiovascular disease (CVD) - and all-cause mortality among patients with non-obstructive coronary artery disease (NOCAD).

**Methods:**

A total of 1046 patients with suspected stable coronary syndrome underwent coronary angiography during 2000–2004, with findings of NOCAD. Serum levels of 8 selected carnitine metabolites were analyzed through liquid chromatography tandem mass spectrometry. Associations with CVD- and all-cause mortality were assessed by multivariable Cox regression models.

**Results:**

Median age at inclusion was 57 years. 51.5% were men. During median (25th- 75th percentiles), 14.1 (13.2–15.4) years of follow-up, 5.7% of the participants died from CVD and the incidence of all-cause mortality was 17.3%. Serum acetyl, octanoyl- and palmitoylcarnitine predicted CVD mortality with multivariable HR and 95% CI (per SD increment log transformed) of 1.36 (1.01–1.83), 1.49 (1.15–1.93) and 2.07 (1.49–2.85), p ≤ 0.04, respectively. Higher serum acetyl- and palmitoylcarnitines were also associated with increased risk of all-cause mortality (HR (95% CI): 1.27 (1.01–1.50), and 1.51 (1.26–1.81), p ≤ 0.007. Baseline levels of the precursors trimethyllysine and ƴ-butyrobetaine, carnitine or the odd chained propionylcarnitine and (iso)valerylcarnitine were not associated with adverse outcomes.

**Conclusion:**

Elevated serum even-chained acylcarnitines predicted adverse long-term prognosis in NOCAD. The strongest risk estimates were observed for palmitoylcarnitine, which predicted both CVD- and all-cause mortality after extensive multivariable adjustments. Underlying pathomechanisms should be further elucidated.

## 1 Introduction

A substantial number of patients undergoing invasive coronary angiography (ICA) due to chest pain and suspected ischemic heart disease turn out not to have obstructive stenoses in their epicardial coronary arteries [1]. Though the etiology of symptoms may be heterogeneous, exercise induced ischemia is a frequent finding in such patients [2]. Hence, a majority of patients with chest pain and non-obstructive coronary artery disease (NOCAD) may have impaired function of the epicardial coronary arteries or microcirculation [1]. Myocardial ischemia occurs due to an imbalance between oxygen supply and demand causing metabolic disturbances and ATP depletion [3]. Fatty acids (FAs) constitute a major energy source of the myocardium [4]. The quaternary ammonium compound carnitine is essential for the transport of the long-chained FAs into the mitochondrial matrix, where the β-oxidation takes place [5]. In the human organism, carnitine exists as free active L-carnitine and esterified forms of different chained lengths, collectively referred to as acylcarnitines [5,6]. Excessive as well as dysfunctional mitochondrial FA oxidation (FAO) have been implicated in the pathogenesis of several lifestyle related diseases and may be mirrored by elevated circulating acylcarnitines [[6], [7], [8], [9], [10], [11], [12]]. Further, acylcarnitines may accumulate resulting from incomplete FAO during myocardial ischemia [13]. Experimental studies have suggested that the acylcarnitines have cardiotoxic properties, including adverse effects on the myocardium [14] as well as on the vasculature [15]. We previously demonstrated that high levels of serum acylcarnitines predicted increased risk of CVD mortality and future acute myocardial infarction among patients with chronic coronary syndrome (CCS), of which the majority had obstructive CAD [16]. To the best of our knowledge, previous studies have not evaluated the prognostic implications of serum acylcarnitines levels specifically in NOCAD. As compared to obstructive CAD, NOCAD has traditionally been linked to a more favorable prognosis [1]. However, recent studies have revealed substantial heterogeneity in long-term CVD risk in NOCAD patients [17]. Hence, there is considerable interest in extending current knowledge on disease mechanisms as well as improving the identification of high-risk phenotypes. We measured circulating carnitine, its precursors trimethyllysine (TML) and ƴ - butyrobetaine as well as five selected carnitine esters. Acetyl-octanoyl-and palmitoylcarnitine represented short-, medium-, and long even chained acylcarnitines, respectively. The odd-chained acylcarnitines were represented by propionyl-and (iso)valerylcarnitines, which are derived from branched-chained amino acids previously being linked with risk of diabetes [18]. In the present study, these 8 metabolites were evaluated in relation to long-term risk of CVD- and all-cause mortality risk among patients with NOCAD.

## 2 Methods

### 2.1 Study population and baseline characteristics

A more detailed description of the study population and the collection of clinical and demographic baseline information are reviewed elsewhere [9,16]. The present work derives from The Western Norway Coronary Angiography Cohort (WECAC). WECAC included 4164 patients with chest pain and suspected CCS who underwent ICA at Stavanger or Haukeland University hospitals in Western Norway during 2000–2004. Our sub-study includes 1046 patients with NOCAD, defined as the findings of no visible or minor atherosclerotic plaques with <50% luminal narrowing at ICA. Segment involvement score (SIS) was calculated as the total number of coronary artery segments exhibiting non-obstructive plaques, following a modified 18-segment American Heart Association model [19]. Information on SIS was available in 764 (73%) of the patients. A total of 667 (63.8%) patients underwent ventriculography, from which information was collected on left ventricular end diastolic pressure (LVEDP). Data on left ventricular ejection fraction (LVEF) was derived from transthoracic echocardiography and was complete in all participants.

Patient-administered questionnaires provided information about medical history, CVD risk factors and current medications. Diagnoses of hypertension and diabetes mellitus were defined by self-report and checked against hospital medical records. Fasting conditioning was defined as > 6 h since consuming any nutrients or fluids [20]. Instructed personnel collected venous blood samples before or right after the ICA. Serum samples were frozen immediately after collection and stored at −80 °C until analyzed in 2009. Serum concentrations of TML, ƴ-butyrobetaine, free carnitine, the even chained acetylcarnitine, octanoylcarnitine, and palmitoylcarnitine; and the branched chained propionylcarnitine and (iso)valerylcarnitine were measured by liquid chromatography-tandem mass spectrometry as previously described [9,16]. The methods for the measurements of estimated GFR, HbA1c, serum apolipoprotein A1, apolipoprotein B and C-reactive protein have also been reported in detail elsewhere [21]. All participants provided written informed consents. The study has been approved by the Norwegian Data Protection Authority and the Regional Committee for Medical and Health Research Ethics (REK 2012/2167 and REK 2013/2324) and was performed according to the declaration of Helsinki. The dataset analyzed during the current study is available from the corresponding author upon reasonable request.

### 2.2 Clinical endpoints and follow-up

Information on CVD- and all-cause mortality during follow-up was collected by linkage to the Norwegian Cause of Death Registry at the Norwegian Institute of Public Health (www.fhi.no/en/hn/health-registries/cause-of-death-registry).

Since we were particularly interested in the relations of carnitine metabolites to CVD risk in NOCAD patients, we defined CVD mortality as the primary endpoint. This outcome included causes of death coded I00–I99 according to the International statistical Classification of Diseases, 10th version (ICD-10). We additionally evaluated all-cause mortality and non-cardiovascular disease mortality as secondary endpoints. The follow-up of the participants lasted until the occurrence of a fatal incident, or no later than December 31st^,^ 2017.

### 2.3 Statistical analysis

The baseline continuous and categorical variables were reported as medians (25th-75th percentiles) or counts (percentages), as appropriate. Relations of the carnitine precursors and esters with relevant clinical baseline characteristics were assessed by Spearman rank correlation analyses. We evaluated associations to CVD- and all-cause mortality using Cox regression models. Hazard ratios (HRs) and 95% confidence intervals (CIs) are reported per SD increment of (log transformed) serum metabolite concentrations. The simple model includes adjustments for age and sex. The multivariable model was additionally adjusted for BMI (continuous), systolic blood pressure (continuous), self -reported smoking status (current, former, never), estimated GFR (continuous), HbA1c (continuous), apolipoprotein A1 (continuous), and apolipoprotein B (continuous). Since we previously have demonstrated that the levels of carnitine precursors and metabolites may show slight variation between fasting and non-fasting subjects [9], we also included fasting status (dichotomous) into the multivariable model. To test the assumption of proportional hazards, we inspected survival plots and calculated scaled Schoenfeld residuals. We generated Kaplan Meier survival curves according to quartiles of serum carnitine metabolites. The continuous relationship of carnitine metabolite levels with all-cause and CVD mortality were visualised by generalized additive regression plots, in which the biomarkers were modelled with a 4 df smoothing spline fit in multivariable Cox regression models.

By determining continuous net reclassification improvement (NRI >0) [22], we evaluated whether the carnitine metabolites improved risk classification of NOCAD patients. We explored model discrimination by areas under the receiver operating characteristic curve (ROC-AUC).

All probability values are 2-tailed with a significance level generally set to 0.05 with the exception of the correlation analyses which, due to multiple testing, where considered significant when <0.001. The statistical analyses were performed using the software packages IBM SPSS Statistics Version 26 (Armonk, NY: IBM Corp) and R version 4.0.4 for Windows (The R Foundation for Statistical Computing, Vienna, Austria, 2016, https://www.R-project.org).

## 3 Results

### 3.1 Baseline characteristics

Baseline characteristics of the study population are presented in Table 1. Median (25th- 75th percentiles) age at inclusion was 57 (51–65) years, 539 (51.5%) of the participants were men and median (25th- 75th percentiles) BMI was 26 [[23], [24], [25], [26], [27], [28]] kg/m2. A total of 428 (40.9%) had hypertension, 75 (7.2%) had diabetes mellitus and 250 (24.0%) were current smokers. Median (25th- 75th percentile) values were for LVEF 70 (65–70)%, LVEDP 16 [[12], [13], [14], [15], [16], [17], [18], [19], [20]]mm Hg and SIS 0 (0–1) segments.

**Table 1:** Baseline characteristics of the study participants (n = 1046).

**Demographics and risk factors**		
Age	57	(51–65)
Male sex	539	(51.5%)
Body mass index, kg/m^2^	26	(23–28)
Smoking	250	(24.0%)
Hypertension	428	(40.9%)
Diabetes mellitus	75	(7.2%)
Fasting status	228	(21.8%)
**Lab**		
s-Apo A1, g/L	1.40	(1.22–1.58)
s-Apo B, g/L	0.90	(0.75–1.07)
s-Triglycerides, mmol/L	1.4	(1.0–2.0)
p-Glucose, mmol/L	5.5	(4.9–6.3)
b-HbA1c mmol/mol	44	(37–52)
s-CRP mg/L	1.67	(0.78–3.50)
s-Creatinine (mmol/L)	84	(77–93)
Estimated GFR, (ml/min/1.73m^2)^	93	(81–102)
s-Trimethyllysine, (μmol/L)	0.63	(0.51–0.80)
s- ʏ-Butyrobetaine, (μmol/L)	0.97	(0.84–1.13)
s- L-carnitine, (μmol/L)	38.6	(34.5–43.3)
s-Acetylcarnitine (C2), (μmol/L)	5.69	(4.58–7.19)
s-Propionylcarnitine (C3), (μmol/L)	0.41	(0.33–0.51)
s-(Iso)valerylcarnitine (C5), (μmol/L)	0.11	(0.082–0.14)
s-Ocatanoylcarnitine (C8), (μmol/L)	0.13	(0.089–0.094)
s-Palmitoylcarnitine (C16), (μmol/L)	0.080	(0.066–0.094)
**Medication**		
Aspirin	578	(55.3%)
Statins		558
Beta blockers		523
Calcium channel blockers	179	(17.1%)
**Clinical endpoints**		
Cardiovascular mortality	60	(5.7%)
Non-cardiovascular mortality	121	(11.6)
All-cause mortality	181	(17.3%)

Continuous variables are presented as median (25th-75th percentile).

Categorical variables are presented as no. (%).

Correlations of carnitine precursors and metabolites with important clinical baseline characteristics have previously been reported for the source population (n = 4164) [16] and similar associations were found in the present sub-study. The metabolites were generally positively inter correlated, and the strongest associations were found between the even chain acylcarnitines (rho = 0.49–0.56, p < 0.001).

TML, acetylcarnitine, octanoylcarnitine and palmitoylcarnitine were positively correlated with age (rho = 0.12–0.20, p < 0.001). In general, positive correlations were seen in relation to BMI, to which the strongest associations were found for the odd-chained proprionylcarnitine and (iso)valerylcarnitine (rho = 0.23 and 0.28, p < 0.001). Acetylcarnitine, octanoylcarnitine and palmitoylcarnitine levels correlated positively with systolic blood pressure (rho 0.14–0.19, p < 0.001). With the exception of TML which showed a weak positive association (rho = 0.13, p < 0.001), the carnitine precursors and esters were not associated with diastolic blood pressure, nor were they significantly correlated with the SIS, LVEF or EDP (rho ≤0.07, p ≥ 0.06).

### 3.2 Baseline levels of carnitine precursors and metabolites in relation to mortality during follow up

**Fig. 1:**
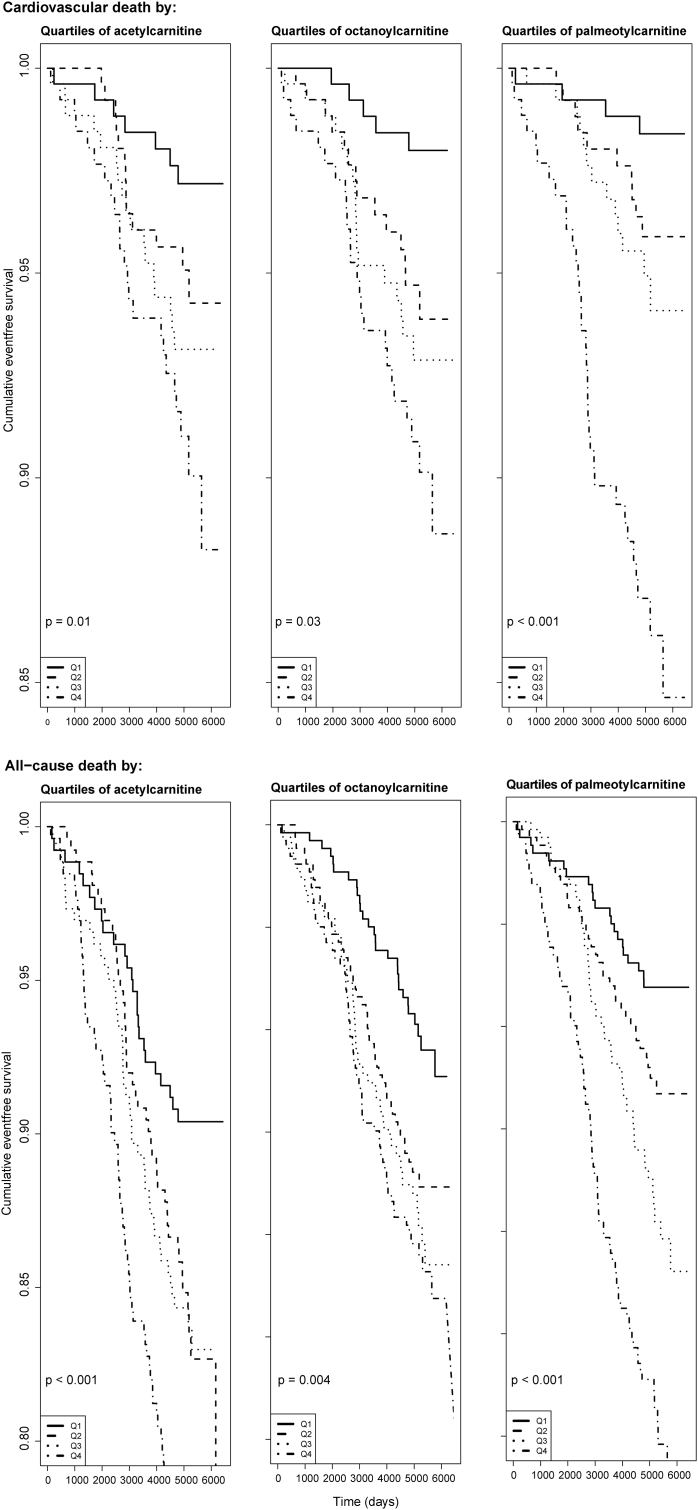
Kaplan-Meier curves showing unadjusted cumulative incidence of clinical events according to quartiles of serum acetylcarnitine, octanoylcarntine and palmitoylcarnitine. Reported p-values are for the corresponding log rank tests.

**Fig. 2:**
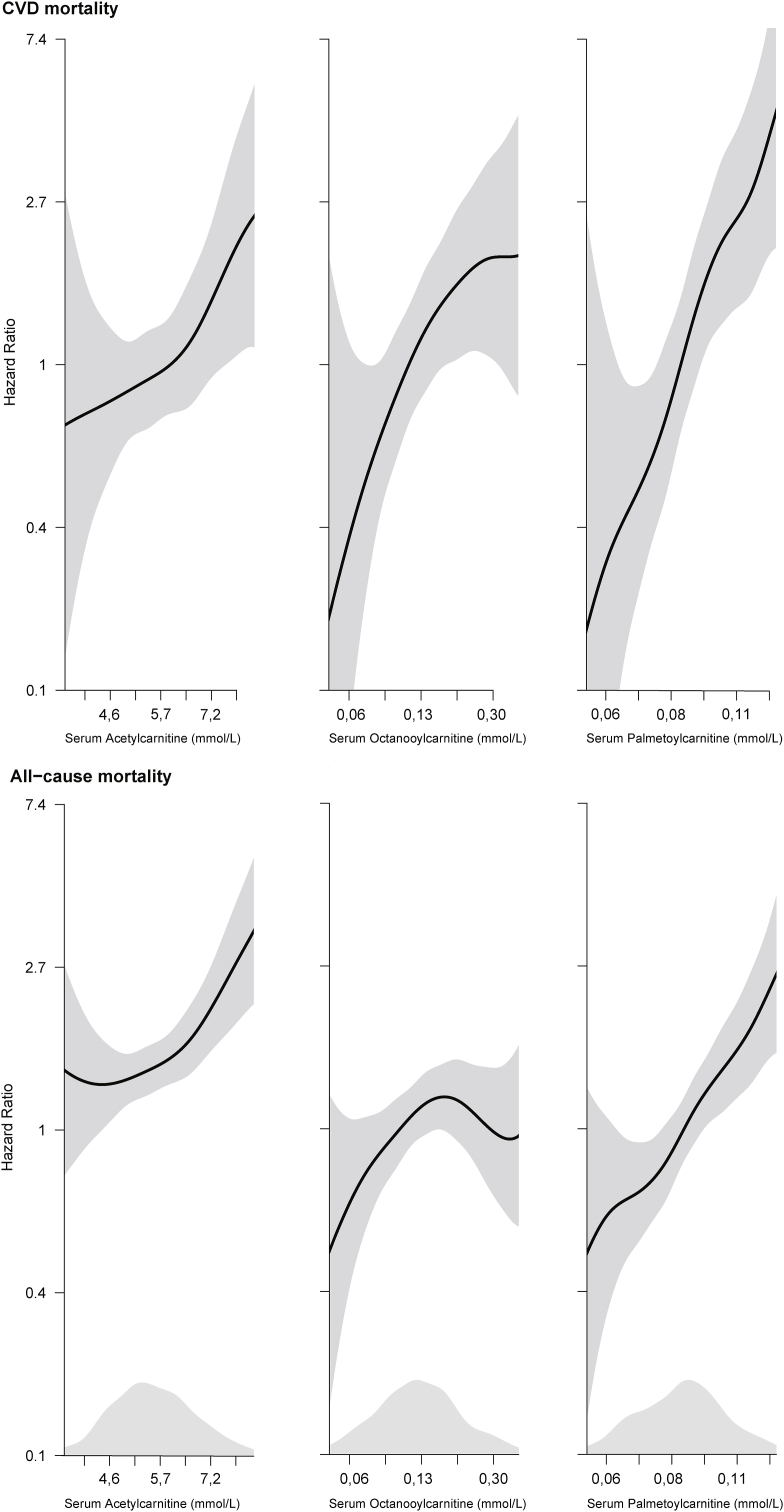
Dose–response relationship between serum acetylcarnitine, octanoylcarnitine and palmitoylcarnitine and risk of clinical events during follow-up obtained by generalized additive regression. The models are adjusted for age, sex, body mass index, systolic blood pressure, smoking, estimated GFR, HbA1c, apoliprotein A1, apolipoprotein B and fasting status. The solid lines show the HR and the shaded areas show 95% confidence intervals. Ranges from the 2.5th to the 97.5th percentiles of exposure variables are included. Density plots show the distribution of acylcarnitines.

During median (25th, 75th percentiles) 14.1 (13.2, 15.4) years of follow-up, 60 (5.7%) of the study participants died from CVD and all-cause mortality occurred in 181 (17.3%). Fig. 1 demonstrates the Kaplan Meier survival curves for quartiles of acetylcarnitine, octanoylcarnitine and palmitoylcarnitine in relation to CVD-and all-cause mortality. Fig. 2 visualizes the multivariable adjusted continuous association of the same metabolites with risk of clinical outcomes during follow-up.

In the primary Cox model, adjusting for age and sex, acetyl-, octanoyl- and palmitoylcarnitine predicted CVD mortality with HR (95% CI) per SD increment of 1.39 (1.07–1.81), 1.52 (1.18–1.94) and 1.78 (1.35–2.34), respectively (p ≤ 0.02). These metabolites also remained significant predictors after multivariable adjustments with HRs (95% CIs) of 1.36 (1.01–1.83), 1.49 (1.15–1.93) and 2.07 (1.49–2.85), p ≤ 0.04 for acetyl-, octanoyl- and palmitoylcarnitine, respectively. Table 2, upper panel).

**Table 2:** Hazard ratio per 1 SD increment of carnitine precursors and esters in relation to long term risk of CVD- and all-cause mortality.

*Panel A) CVD mortality*	Adjusted for age and sex				Multivariable*		
	**Hazard ratio**		**95% CI**	**P**	**Hazard ratio**	**95% CI**	**P**
Trimethyllysine	1.10	0.85–1.41		0.47			
ʏ-Butyrobetaine	0.99	0.75–1.32		0.97			
Carnitine	1.00	0.76–1.32		0.99			
Acetylcarnitine	1.39	1.07–1.81		0.02	1.36	1.01–1.83	0.04
Propionylcarnitine	1.12	0.84–1.48		0.45			
(Iso)valerylcarnitine	0.98	0.74–1.28		0.86			
Octanoylcarnitine	1.52	1.18–1.94		0.001	1.49	1.15–1.93	0.003
Palmotoylcarnitine	1.78	1.35–2.34		<0.001	2.07	1.49–2.85	<0.001

***Panel B) All-cause mortality***	**Adjusted for age and sex**				Multivariable*		
	**Hazard ratio**		**95% CI**	**P**	**Hazard ratio**	**95% CI**	**P**
Trimethyllysine	1.05	0.91–1.22		0.50			
ʏ -Butyrobetaine	0.88	9.75–1.03		0.11			
Carnitine	1.02	0.87–1.19		0.83			
Acetylcarnitine	1.33	1.14–1.55		<0.001	1.27	1.01–1.50	0.007
Propionylcarnitine	0.99	0.85–1.16		0.89			
(Iso)valerylcarnitine	1.14	0.99–1.30		0.07			
Octanoylcarnitine	1.18	1.01–1.36		0.03	1.13	0.96–1.34	0.14
Palmitoylcarnitine	1.45	1.24–1.70		<0.001	1.51	1.26–1.81	<0.001

*Adjusted for age, sex, BMI, systolic blood pressure, smoking, estimated GFR, HbA1c, ApoA1, ApoB and fasting status.

Similar patterns, although with numerically weaker risk estimates, were observed in relation to all-cause mortality. Acetylcarnitine, octanoylcarnitine and palmitoylcarnitine were all significant predictors in age and gender adjusted analyses. In multivariable analyses, the associations remained statistically significant for acetylcarnitine and palmitoylcarnitine only (Table 2, lower panel). As shown in Supplemental Table 1, palmitoylcarnitine was the only metabolite being significantly associated with non-cardiovascular disease mortality in multivariable analyses. Notably, the risk estimate was numerically weaker than that observed for CVD mortality.

Baseline levels of the precursors TML and ƴ-butyrobetaine, carnitine or the odd chained propionylcarnitine and (iso)valerylcarnitine were not associated with clinical outcomes during follow-up.

### 3.3 Reclassification and discrimination analyses

Palmitoylcarnitine improved risk classification for CVD mortality, with a NRI (95% CI) of 0.45 (0.19–0.70), P ≤ 0.001, when added to the multivariable model. In addition, both acetylcarnitine and palmitoylcarnitine provided statistically significant NRIs (95% CI) of 0.18 (0.02–0.35) and 0.34 (0.18–0.51) in relation to all-cause mortality (P < 0.03, Supplemental Table 2).

ROC-AUC for the multivariable model without carnitine biomarkers were 0.784 and 0.787 for CVD and all-cause mortality, respectively. Palmitoylcarnitine was the only metabolite providing a significant increment in ROC-AUC for CVD mortality (Δ0.030, p = 0.03). None of the biomarkers significantly increased the ROC-AUC for all-cause mortality (p ≥ 0.06).

## 4 Discussion

### 4.1 Principal findings

In this prospective cohort study including a total of 1046 patients with chest pain and NOCAD, we demonstrated associations of the even-chained acetylcarnitine, octanoylcarnitine and palmitoylcarnitine with long-term prognosis. A particularly strong dose-response relationship was seen for palmitoylcarnitine, which predicted both CVD- and all-cause mortality after extensive multivariable adjustment. Palmitoylcarnitine also improved risk classification for CVD- and all-cause mortality as well as increased model discrimination for CVD mortality. Baseline levels of the precursors TML and ƴ-butyrobetaine, carnitine or the odd chained propionylcarnitine and (iso)valerylcarnitine were not associated with adverse outcomes.

### 4.2 Carnitine metabolism

Carnitine can be ingested from the diet or synthesized from TML, with ƴ-butyrobetaine, as an intermediate product. In its free active form, L-carnitine plays an essential role in the metabolism of free FAs. The FAs are transported into the cells through strictly regulated transport systems [5] where they, in the cytosol, combine with acyl-coenzyme A (acyl-CoA) to form esters of different chained lengths [5,6]. The long-chained acyl-CoAs are incapable of diffusion through the mitochondrial membrane. During shuttling, the acyl group merges with carnitines to form acylcarnitines, which are actively transported, into the mitochondrial matrix, where they are transformed back into free carnitine and acyl-CoAs. The latter goes through β-oxidation in the tricarboxylic acid cycle [5,23] and into ATP production. Long and medium chained carnitine esters are partially β-oxidized in peroxisomes, before complete β-oxidation in the mitochondria [24].

### 4.3 Acylcarnitine accumulation and lipotoxicity

Long chained FAs are the main energy source of the cardiomyocytes, constituting more than 70% of the heart's total energy supply. Under normal physiological circumstances, there is a strict metabolic balance between the FA uptake and the β-oxidation in the cardiomyocytes [4]. However, when there is a mismatch between cellular FA uptake and FAO, an intracellular accumulation of FA metabolites emerges causing lipotoxicity [14]. Acylcarntines can accumulate due to increased substrate availability in obesity [14], insulin resistance and diabetes mellitus [14,25]. An excess of acylcarnitines may also be secondary to inhibited FAO due to aging [14,26] or in myocardial ischemia and reperfusion [13].

Accumulation of acylcarnitines may give rise to increased formation of cardiotoxic reactive oxygen species through mitochondrial membrane hyperpolarization [27]. Oxidative stress may generate endothelial disturbances, inflammation and accumulation of intimal vascular smooth muscle cells, which in turn seem to be driving forces in the development of atherosclerosis [28]. Interestingly, a recent study also revealed that the carnitine axis may be implicated in arterial calcification [19].

Besides their proposed effects in atherogenesis, long-chained acylcarnitines seem to have an impact on cardiac electrophysiological and mechanical processes through electrolyte disturbances and a destabilization of the action potential and depolarization. Hence, experimental models have suggested that carnitine esters may mediate adverse effects on cardiac contractility as well as having pro-arrhythmic properties [29].

### 4.4 The carnitine axis and cardiometabolic disease

Serum acylcarnitine profiles have been extensively studied as potential biomarkers of adverse cardiometabolic prognosis. In cross sectional studies, elevated levels of plasma long-chained acylcarnitines were found in patients with obesity [30], insulin resistance [31], and type 2 diabetes [32]. Both short and long-chained carnitine esters were associated with increased risk of type 2 diabetes development in prospective and nested case control studies [7,10]. We previously identified serum levels of TML, ƴ-butyrobetaine, and palmitoylcarnitine as predictors of incident type 2 diabetes among patients with CCS [9]. Acylcarnitine profiles have also been linked to increased risk of CVD mortality among patients with type 2 diabetes [6], possibly mirroring a cardiotoxic accumulation of FAs due to an insufficient FAO. Increased levels of palmitoylcarnitine have been associated with adverse prognosis among patients with heart failure [33]. Recently, elevated levels of several medium- and long-chained acylcarnitines were linked to increased risk of atrial fibrillation [34]. Furthermore, arrhythmias have been observed in children with FAO disorders resulting in the accumulation of acylcarnitines [35]. We previously demonstrated that high levels of even chained acylcarnitines predicted increased risk of CVD mortality and future acute myocardial infarction among patients with CCS, of which the majority had obstructive CAD [16]. We now reveal similar associations to CVD mortality and all-cause mortality among patients with NOCAD, indicating that the adverse prognostic impact is not mediated by obstructive CAD progression. In the present study, we found no significant risk association between the precursor TML and adverse outcomes. However, we previously demonstrated that higher serum TML levels predict angiographic advancement of atherosclerosis among patients with obstructive CAD [36]. Additionally, elevated TML was associated with incident acute myocardial infarction in patients with obstructive CAD [37]. These findings may indicate that serum TML, in contrast to the even chained acylcarnitines, is primarily a risk predictor in patients with obstructive atherosclerosis.

### 4.5 Clinical implications

In our cohort, clinically stable patients with chest pain and suspected myocardial ischemia, but without invasively confirmed obstructive CAD, had overall a rather favorable CVD prognosis. Still, we identified palmitoylcarnitine as a significant predictor of CVD mortality during median 14 years of follow-up. The strong predictive value of palmitoylcarnitine in the absence of obstructive CAD suggest that the risk association may not be due to atherosclerotic plaque progression. However, the underlying pathomechanisms of our findings are not clear. The carnitine axis has been linked to vascular and myocardial dysfunction as well as pro-arrhythmic properties in previous experimental studies [15,29,33]. We observed significant correlations of the even chained acylcarnitines to BMI and systolic blood pressure. In contrast, no clear associations were found to LVEF or LVEDP as measures of systolic and diastolic function, respectively. Since the event rate for CVD mortality was overall low in our cohort, we did not have power to address subtypes like fatal atherosclerotic events, arrhythmic events or heart failure, which warrant more investigations. Our findings also should motivate future clinical studies aimed at discerning how the carnitine axis correlate with more sensitive measures of myocardial function, vascular function and ischemia, as assessed by novel echocardiographic and magnetic resonance tomography techniques. Moreover, the role of serum long-chained acylcarnitines for the identification of high-risk phenotypes in NOCAD should be further explored.

### 4.6 Strengths and limitations

Major strengths of our study include the large sample size, prospective design, long follow-up time and detailed descriptions of clinical and biochemical baseline characteristics. The information on clinical events during follow-up was collected from the Norwegian Cause of Death Registry to which reporting is mandatory for all deaths in Norway. We cannot exclude that some misclassification may have occurred, but we do not suspect any misclassification of endpoints vary according to the levels of metabolites. Hence, a bias in this regard is unlikely.

Since the ICAs were performed with the main aim of identifying obstructive CAD and not specifically to evaluate NOCAD, the SIS may possibly be under reported. Further, coronary computed tomography angiography (CCTA) would usually be the first diagnostic test for the evaluation of chest pain and suspected myocardial ischemia in current clinical practice, but was not in routine use at our hospital during study recruitment. CCTA is more sensitive than ICA for the detection of atherosclerosis [38] and may have provided more information on the association of carnitine metabolites with the extension and morphologies of atherosclerotic plaques as well as with coronary artery calcification. Due to the observational nature of our work, we cannot conclude whether the carnitine metabolites as such mediate adverse health effects or only represent passive biomarkers of the disease process. A replication cohort was not available for the present cohort. Moreover, although of statistical significance, the improvements in discrimination and risk classification were modest, and of uncertain clinical relevance. Hence, the potential role of long-chained acylcarnitines as prognostic biomarkers in NOCAD needs further exploration.

### 4.7 Conclusion

Among patients with chest pain of suspected ischemic etiology, but without obstructive CAD at ICA, elevated serum levels of even chained acylcarnitines were associated with impaired long-term prognosis. The strongest risk associations were observed for the long-chained palmitoylcarnitine, which predicted both CVD mortality and all-cause mortality. Future studies should further elucidate the pathogenic significance of dysfunctional FA metabolism and acylcarnitine accumulation in the mediation of CVD risk in NOCAD.

## Funding

The study was funded by the 10.13039/501100010698Norwegian Heart and Lung Patient Organisation, the Norwegian 10.13039/501100003506Ministry of Health and Care Services, the 10.13039/501100004257Western Norway Regional Health Authority and the Department of Heart Disease, 10.13039/501100010586Haukeland University Hospital, Bergen, Norway. None of the study sponsors were involved in study design, data collection, analysis and interpretation of data, writing, or in the decision to submit the paper.

## Contribution statement

S.K.S., I.K and E.R.P. analysed and interpreted data, and wrote the manuscript. E.S., M.T.L and G.F.T.S made substantial contributions to analysis and interpretation of data. R.K.B. and A.S. supervised the analyses of carnitine precursors and ester and interpreted the data. E.R.P., G.F.T.S., and O.K.N. collected and processed clinical data. O.K.N. and E.R.P. conceived and designed the study. S.K.S., I.K., E.S., A.S., M.T.L, R.K.B., G.F.T.S, O.N., and E.R.P. edited and revised the manuscript. S.K.S. is the guarantor of this work and, as such, had full access to all the data in the study and takes responsibility for the integrity of the data and the accuracy of the data analysis. All authors have approved the final version of the article.

## Declaration of competing interest

The authors declare that there is no conflict of interest associated with this manuscript.
