# Supplementary material for: Even chained acylcarnitines predict long-term cardiovascular prognosis in patients with chest pain and non-obstructive coronary artery disease
Source: Int J Cardiol Cardiovasc Risk Prev. 2022 May 17;14:200134. doi: 10.1016/j.ijcrp.2022.200134 (PMC9136115; doi:10.1016/j.ijcrp.2022.200134)
Supplement: Multimedia component 1 [file mmc1.docx]

***Supplemental Table 1.* *Hazard ratio per 1 SD increment of carnitine precursors and esters in relation to long term risk of non-cardiovascular disease mortality***

**Adjusted for age and sex Multivariable***

**Hazard ratio 95% CI P Hazard ratio 95% CI P**Trimethyllysine 1.03 0.86-1.01 0.06
ʏ-Butyrobetaine 0.83 0.68-1.32 0.97
Carnitine 1.03 0.85-1.25 0.78
Acetylcarnitine 1.30 1.08-1.57 0.006 1.22 0.99-1.50 0.07
Propionylcarnitine 0.93 0.77-1.13 0.48
(Iso)valerylcarnitine 0.98 0.74-1.28 0.86
Octanoylcarnitine 1.21 1.04-1.4| 0.015 0.96 0.78-1.17 0.26
Palmotoylcarnitine 1.31 1.09-1.59 0.005 1.29 1.04-1.61 0.02
*Adjusted for age, sex, BMI, systolic blood pressure, smoking, estimated GFR, HbA1c, ApoA1, ApoB and fasting status
