# Supplementary material for: Even chained acylcarnitines predict long-term cardiovascular prognosis in patients with chest pain and non-obstructive coronary artery disease
Source: Int J Cardiol Cardiovasc Risk Prev. 2022 May 17;14:200134. doi: 10.1016/j.ijcrp.2022.200134 (PMC9136115; doi:10.1016/j.ijcrp.2022.200134)
Supplement: Multimedia component 2 [file mmc2.docx]

***Supplemental Table 2. Net reclassification improvement (NRI)***

**CVD mortality**

***Multivariable model** NRI 95% CI P**

Without biomarker

With acetylcarnitine 0.24 -0.02-0.51 0.07

With octanoylcarnitine 0.21 -0.06-0.29 0.13

With palmitoylcarnitine 0.45 0.19-0.70 <0.001

**All-cause mortality**

***Multivariable model** NRI 95% CI P**

Without biomarker

With acetylcarnitine 0.18 0.02-0.35 0.03

With octanoylcarnitine 0.08 -0.08--0.17 0.33

With palmitoylcarnitine 0.34 0.18-0.51 <0.001

*Adjusted for age, sex, BMI, systolic blood pressure, smoking, estimated GFR, HbA1c, apoA1, apoB and fasting status
